# Widespread glacier advances across the Tian Shan during Marine Isotope Stage 3 not supported by climate-glaciation simulations

**DOI:** 10.1016/j.fmre.2022.01.033

**Published:** 2022-03-02

**Authors:** Qing Yan, Lewis A. Owen, Chuncheng Guo, Zhongshi Zhang, Jinzhe Zhang, Huijun Wang

**Affiliations:** a Nansen-Zhu International Research Centre, Institute of Atmospheric Physics, Chinese Academy of Sciences, Beijing 100029, China; b Key Laboratory of Meteorological Disaster/Collaborative Innovation Center on Forecast and Evaluation of Meteorological Disasters, Nanjing University of Information Science and Technology, Nanjing 210044, China; c Department of Marine, Earth, and Atmospheric Science, North Carolina State University, Raleigh, NC 27695, United States; d NORCE Norwegian Research Centre, Bjerknes Centre for Climate Research, Bergen, Norway; e Department of Atmospheric Science, School of Environmental Studies, China University of Geosciences, Wuhan 430074, China

**Keywords:** Glacier modeling, Glacier sensitivity, MIS 3, MIS 2, Tian Shan, Paleoclimate modeling

## Abstract

Whether there were more extensive glaciations during the Marine Isotope Stage (MIS) 3 relative to MIS 2 across the Tian Shan in Central Asia is intensely debated because of the uncertainty in chronological data and fully understanding the driving mechanisms. To help resolve the ongoing debate, we assess the climate sensitivity of the glaciers and reconstruct the extent of glaciation during MIS 2 and 3 across the Tian Shan, using a glacier-resolving (250 × 250 m) ice sheet model asynchronously coupled with a global climate model. Our results demonstrate that the equilibrium-line altitude (ELA) over the Tian Shan decreases by ∼180 m for every 1 °C cooling under a modern precipitation regime, but precipitation reduction greatly lowers the sensitivity of the glaciers to temperature decrease (e.g., the effect of 2 °C cooling is broadly offset by a 50% decrease in precipitation). Under the modeled colder/drier-than-present climate, the model predicts an ELA depression (∆ELA) of ∼75 m (162 m) over the Tian Shan during MIS 3 interstadials (stadials). The extent of MIS 3 glaciation is much smaller than that during MIS 2 (i.e., ∆ELA = ∼726 m). The more extensive glaciation during MIS 2 is largely attributed to the enhanced summer cooling. Furthermore, through a site-to-site model-data comparison, we find that the closest match between the modeled glacier margin and the locations of the glacial deposits previously argued to be MIS 3 is generally achieved under MIS 2 climatic conditions. These results suggest more extensive glacier advances over the Tian Shan during MIS 2 than MIS 3 on a regional scale, although MIS 3 glaciation may still occur in individual glacier catchments. This pattern suggests general synchronicity with the timing of maximum Northern Hemisphere ice sheets during the last glacial, which should be further tested in a multimodel framework in the future.

## Graphical abstract

**Figure:**
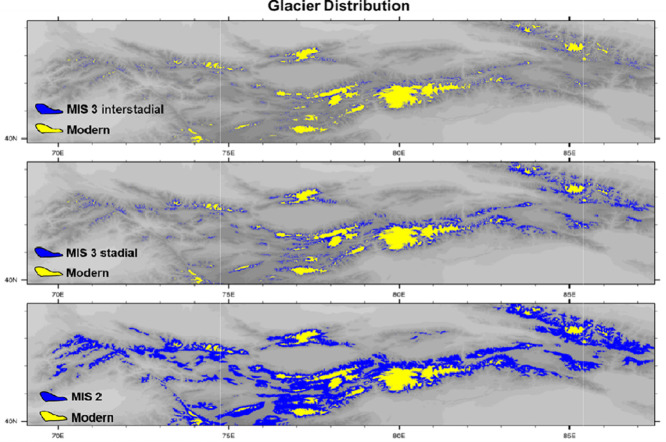

## 1 Introduction

Past glacier fluctuations offer the potential to understand variations in past climate and landscape over Earth's elevated regions [1]. High-mountain Asia, mainly the Himalaya, Tibet, the Pamir, and the Tian Shan, has the largest number of contemporary glaciers outside the polar regions and abundant glacial landforms/sediments that provide records of past glacier oscillations to decipher the spatiotemporal pattern of climate change. Although glacial geologic evidence [2], [3], [4] points to continental-scale glaciations at the high latitudes of the Northern Hemisphere during Marine Isotope Stage (MIS) 2 (∼29–14 ka [5]), little evidence supports the development of an ice sheet over High-mountain Asia at that time. Instead, there is increasing evidence for alpine-style glaciation, e.g., with extensive valley glaciers and expanded ice caps, over High-mountain Asia during MIS 2 [6], [7], [8], [9].

**Fig. 1:**
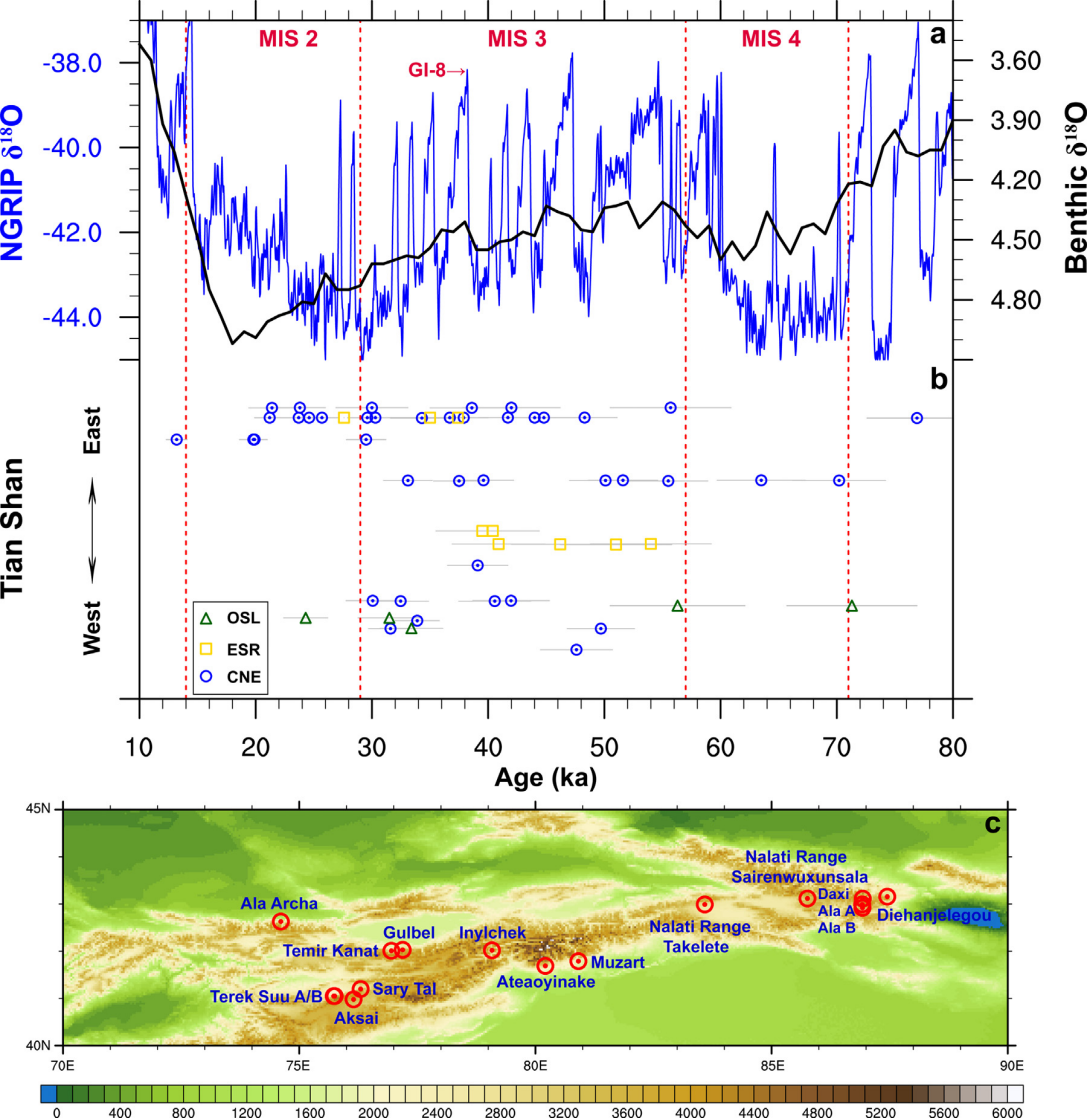
(a) The evolution of δ^18^O from the North Greenland Ice Core Project (NGRIP) ice core (‰; [40]) and the benthic δ^18^O (‰; [5]). (b) The chronological dataset of the major MIS 3 glacier advances proposed for the Tian Shan [13,23,24] based on cosmogenic nuclide exposure (CNE), optically stimulated luminescence (OSL), and electron spin resonance (ESR) dating. Each dot represents the exposure age and associated dating uncertainty of glacial moraine. The chronological dataset clearly shows a widespread glaciation over the Tian Shan during MIS 3. (c) The locations of the previously argued MIS 3 glaciation in (b) over the Tian Shan.

Notably, the timing of the most extensive glaciation is not synchronous across High-mountain Asia, with growing evidence for glaciers reaching their maximum extents predating MIS 2 in many mountain areas [6,7,[10], [11], [12]]. In particular, there are many studies suggesting a major glaciation during MIS 3 (∼57–29 ka) or earlier instead of MIS 2 over the Tian Shan [13], [14], [15], [16], [17], [18], [19], [20], [21], [22], [23], which is out of phase with the change in the global ice volume (Fig. 1). However, the view of extensive MIS 3 glaciation in the Tian Shan has been challenged in recent years. Based on 114 ^10^Be ages from 25 moraines across the Tian Shan [8], it is suggested that only one regional glacial stage can be robustly identified, with glacier advances occurring during MIS 2. Gribenski et al [24]. critically assessed the chronological data of the previously proposed MIS 3 glaciation in Central Asia, suggesting that the interpretation of MIS 3 glaciation remains equivocal. Furthermore, emerging field investigations tend to revise the age of the previously “MIS 3” glacial expansion over the Tian Shan. For example, the ^10^Be exposure ages from the outermost (21.7 ± 7 ka) and upstream (16.0 ± 2.6 ka) moraines over the Muzart valley in the western Tian Shan suggest ice margin oscillations within MIS 2 [23], in sharp contrast to the previous ages (∼40–72 ka) based on electron spin resonance dating [18].

Additionally, the inferred MIS 3 glacier expansions in the Tian Shan have been attributed to significantly wetter conditions at that time, at least relative to MIS 2 [20,25]. However, geologic evidence directly from the Tian Shan indicates extremely drier conditions during MIS 3 and 2 than the present [26], [27], [28]. At the same time, it remains difficult to quantitatively assess the relative aridity between the two periods. Even if MIS 3 experienced wetter conditions relative to MIS 2, a widespread MIS 3 glaciation requires higher glacier sensitivity to precipitation change than temperature decrease. This, in turn, partially implies that ablation should be dominated by sublimation for glaciers over the Tian Shan [29], but modern observations indicate surface ablation is the dominant process and summer temperature controls the fluctuations of glaciers over the Tian Shan [30], [31], [32]. Thus, in terms of glacial chronological data and driving mechanisms, whether the formerly classified MIS 3 glaciation happened across the Tian Shan remains questionable.

To help resolve the ongoing debate on MIS 3 glaciation, we perform a series of climate-glaciation simulations during MIS 3 by combining a glacier-resolving (250 m × 250 m) ice sheet model and a global climate model. Through an evaluation of glacier sensitivity to climate change and detailed data-model comparisons for glaciations during MIS 3, we demonstrate that the Tian Shan may experience more extensive glacier advances during MIS 2 than during MIS 3 on a regional scale, highlighting general synchronicity with the timing of the maximum volume of the Northern Hemisphere ice sheets.

## 2 Methodology

### 2.1 Climate model

We use the fast version of the Norwegian Earth System Model (NorESM1-F) [33] to simulate large-scale climatic change during MIS 3. The NorESM1-F builds on the Community Climate System Model version 4 (CCSM4) and is designed for multi-millennial paleoclimate or large ensemble simulations [34]. The atmospheric and land components of NorESM1-F are based on the Community Atmosphere Model 4 and Community Land Model version 4, respectively. The oceanic component differs from the CCSM4 and is derived from the Miami Isopycnic Coordinate Ocean Model, with the sea ice component from the Los Alamos Sea Ice Model version 4. The horizontal resolution for the atmospheric and land components is ∼2°, with a 1° horizontal resolution for the ocean and sea-ice components. The NorESM has been proven to reasonably reproduce the major features of the modern and past climates on various timescales [34], [35], [36].

### 2.2 Ice sheet model

The Parallel Ice Sheet Model (PISM), a three-dimensional, thermodynamically coupled, polythermal ice-sheet model [37] is utilized to study glacier response to climate perturbations. In PISM, the total glacier mass depends on the balance between accumulation and ablation, which in turn is determined by monthly temperature and precipitation. Specifically, accumulation (i.e., snowfall) is identical to precipitation when the air temperature is below 0 °C and is linearly reduced to zero when the air temperature rises to 2 °C. The ablation is estimated by the positive-degree day scheme. The degree-day factor for ice (snow) is set to 12 mm (4 mm) water equivalent d^–1^ °C^–1^, with a spatially varied standard deviation of surface air temperature derived from High Asia Refined analysis version 2 [38]. Meantime melted ice is allowed to become superimposed ice (by 30%). For ice thermodynamics, PISM uses a hybrid scheme (i.e., the combined shallow ice and shallow shelf approximations) to incorporate the effects of vertical deformation and longitudinal stretching, which enables the simulations of realistic fast-flowing ice streams or outlet glaciers. An enthalpy-based scheme is employed in PISM, which guarantees energy conservation even when the temperature is at the pressure-melting point. Moreover, this scheme accounts for melting and refreezing processes in temperate ice, allowing polythermal and fully-temperate glaciers to be modeled. A “pseudo-plastic” law that relates ice base velocity to the basal shear stress is employed for the estimation of basal sliding. Additionally, glacio-isostatic adjustment is depicted by the modified elastic lithosphere relaxing asthenosphere model. More details concerning the PISM are provided in the supplementary material (Text S1).

Here, we run the PISM at a horizontal resolution of 250 × 250 m (5800 × 1400 grid points) covering the Tian Shan (∼40°–45°N; 70°–90°E) with 51 vertical ice layers and 21 bedrock layers, respectively, giving a computational box in which ∼6 × 10^8^ unknowns are solved for. The present-day topography is derived from the hole-filled seamless SRTM (Shuttle Radar Topography Mission) data version 4 [39] (Fig. S1). The modern air temperature and precipitation are obtained from the High Asia Refined analysis version 2 at a resolution of 10 km [38] (Fig. S1), which is converted to the 250-m resolution via bilinear interpolation, with the topographic effect corrected for temperature (assuming an atmospheric lapse rate of –7.3 °C/km). Preliminary evaluations demonstrate that the 250-m-resolution PISM can satisfactorily reproduce the observed glacier distribution over the Tian Shan (Fig. S2), with a total glacier area of ∼18.3 × 10^3^ km^2^. However, the model is still inefficient to depict very small valley glaciers due to the uncertainty in climatic forcing and the model itself.

### 2.3 Experimental design

MIS 3 is characterized by multiple millennial-scale abrupt climate transitions from colder stadial to warmer interstadial states, each with a gradual return to cold stadial conditions (Fig. 1a; [40]). In this study, we first perform a preindustrial control run and the MIS 3 interstadial experiment. Compared with the preindustrial experiment, orbital parameters for the MIS 3 experiment are set to the levels of 38 ka, immediately following the onset of Greenland Interstadial 8 (Fig. 1a). This leads to increased insolation during spring and summer at the mid-latitudes of the Northern Hemisphere, with reduced insolation during boreal autumn and winter (Fig. S3). CO_2_ concentration is reduced from 285 ppm in the preindustrial to 215 ppm in the MIS 3 interstadial experiment **(**Table S1**)**, together with lower CH_4_ and N_2_O concentrations (i.e., 550 and 260 ppb). The configurations of ice sheets are derived from a data-constrained ice sheet model [41], including the size and height of Antarctica, Greenland, North American, and Eurasian ice sheets. Besides, we modify the land-sea mask according to a lowering of global mean sea-level by 70 m, resulting in the closures of Bering Strait and the Canadian Archipelago. The preindustrial and MIS 3 interstadial experiments are integrated for 2000 and 2500 years, respectively, to reach a quasi-equilibrium climate state. We take the mean climate during the last 100 years of the two experiments to represent the preindustrial and MIS 3 interstadial climates.

We use the freshwater hosing method to force the equilibrium interstadial climate state into a cold stadial-like climate state. In brief, initializing and branched off from the MIS 3 interstadial experiment at the year 1700, we evenly inject a freshwater flux of 0.33 Sv in the North Atlantic (50°–70°N) for 500 years, during which a full stadial-like state is achieved with extensive winter sea ice cover in the Nordic Seas and North Atlantic. Subsequently, the freshwater injection is terminated, and the model is integrated for another 300 years. We take the mean climate during the last 300 years of the freshwater hosing experiment as a reference for the MIS 3 stadial state. More information concerning the experimental design and evaluation of simulation results is given in Guo et al [42]. and Jansen et al [43].

Next, the spatially varied climatic forcing from the NorESM1-F is used to drive the PISM to simulate glacier behavior over the Tian Shan. Given the relatively coarse resolution of the global climate model, we adopt the “anomaly” method to construct the climatic forcing used in PISM. We first calculate the standard anomalies for temperature and fractional anomalies for precipitation (relative to preindustrial) from the NorESM1-F. These anomalies are bilinearly interpolated to the resolution of PISM and are then added to the present-day climatic fields in PISM. The PISM is integrated for >6000 years to reach quasi-equilibrium in terms of ice extent, using the grid refinement technique for computational efficiency [44].

### 2.4 Model outputs for the gLGM

Here, we use the model outputs for the global last glacial maximum (gLGM) from 21 climate models (Table S2) that participate in Paleoclimate Modeling Intercomparison Project (PMIP) Phase 2, 3, and 4 to examine climatic change during MIS 2. The external forcing for the gLGM experiments includes different extents and heights of ice sheets, orbital parameters, land-sea distribution, and greenhouse gas concentrations [45]. Compared with the boundary conditions used in MIS 3, the extent of ice sheets is much larger during MIS 2, especially for the Laurentide Ice Sheet [41]. The gLGM also features a lower CO_2_ concentration than MIS 3 (e.g., 190 *vs.* 215 ppm). However, changes in orbital parameters lead to higher insolation during boreal summer during MIS 3 relative to the gLGM (Fig. S3). Preliminary evaluation indicates that only 10 out of 21 models can reproduce the reconstructed colder and drier climate over the Tian Shan during the gLGM than present (Table S2), which are then used to study climate change during MIS 2.

## 3 Climate sensitivity of glacier over the Tian Shan

**Fig. 2:**
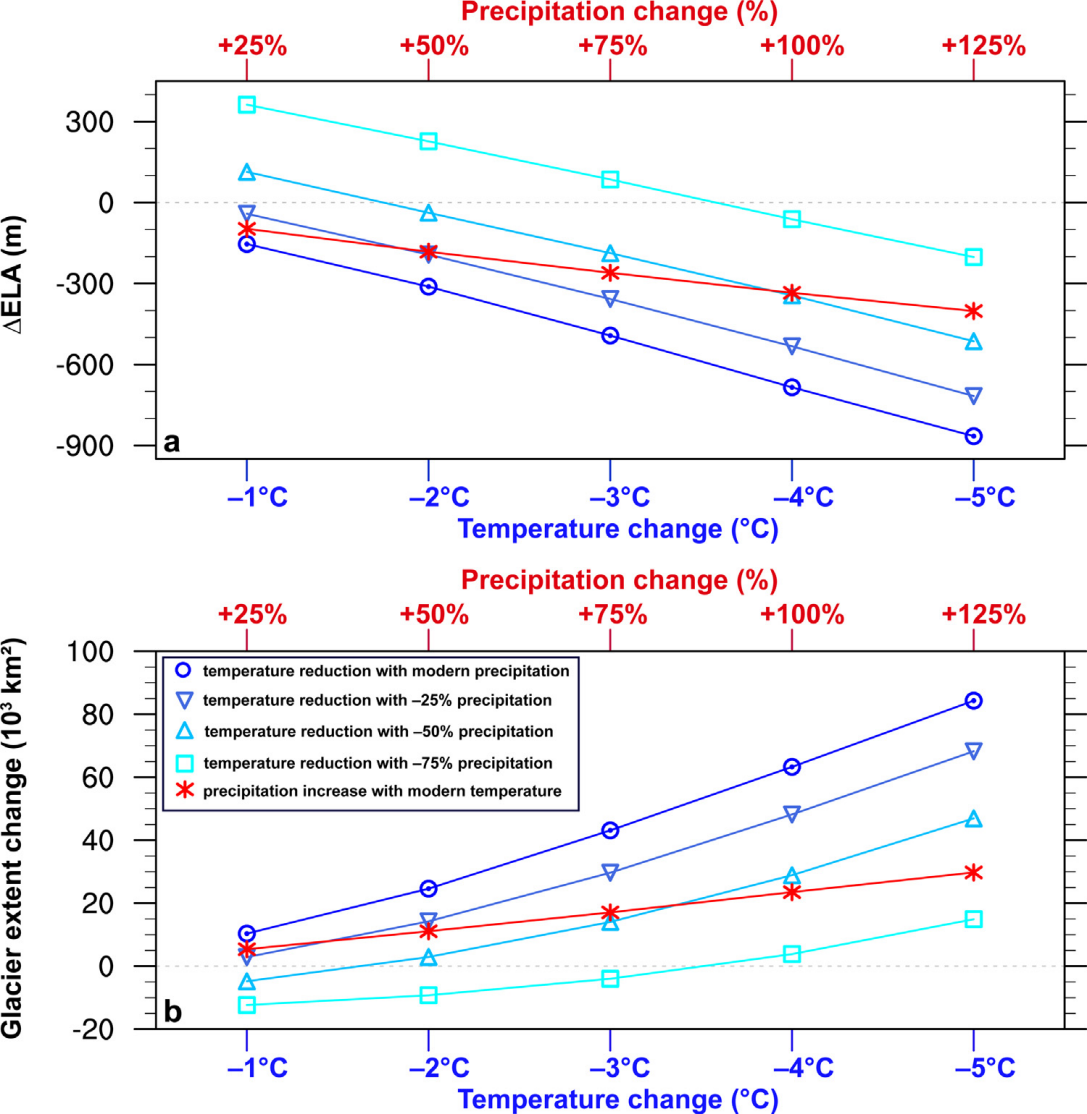
(a) Change in equilibrium-line altitudes (∆ELA; m) relative to present-day over the Tian Shan as a function of the imposed step annual cooling of −1 to −5.0 °C under different precipitation regimes and step precipitation increase of +25% to +125% under a modern temperature regime. (b) Same as (a) but for the total glacier extent (10^3^ km^2^). The ELA here is defined as the altitude where the modeled net mass balance of a glacier is zero. Note that these simulations are performed at a resolution of 500 m.

At the first step, we evaluate the climate sensitivity of glaciers via imposing a stepwise change in temperature (‒1 to ‒5 °C) and in precipitation (+25% to +125%) in PISM, respectively. Under a precipitation regime equal to present-day, the stepwise cooling leads to accelerated glacier expansions over the Tian Shan, increasing the glacierized area by ∼18.7 × 10^3^ km^2^/°C and lowering the equilibrium-line altitude (ELA) by ∼180 m/°C (Fig. 2). Driven by a 5 °C temperature depression, the PISM predicts glaciation of high-altitude catchments and the growth of outlet glaciers into lower valleys across the Tian Shan (Fig. S4), with a total glacier area of ∼102 × 10^3^ km^2^ (∼5 times more extensive than the present-day glacier cover) and an ELA of ∼3182 m (∼865 m lower relative to present). Keeping temperature unchanged, an increasingly wetter climate favors glacier advances, with an expansion of glacier extent by ∼6.1 × 10^3^ km^2^ and an ELA depression (∆ELA) of ∼76 m per 25% more precipitation (Fig. 2).

Furthermore, we investigate how precipitation decrease may regulate glacier sensitivity to temperature decrease, considering that a colder climate is generally associated with reduced precipitation. The results indicate that precipitation decrease greatly lowers the sensitivity of glaciers to temperature decrease (Fig. 2). In response to a cooling of 1‒5 °C, the increasing rate of glacier area reduces by ∼21% to ∼14.7 × 10^3^ km^2^/°C when precipitation is reduced by 50%, and further to ∼8.0 × 10^3^ km^2^/°C when precipitation is reduced by 75%. In terms of total glacier area and ELA, the effect of 4 °C (2 °C) cooling on glaciation is roughly compensated by a decrease of precipitation by 75% (50%) in our simulations (Fig. 2), which is generally consistent with the modeled glacier sensitivity using an energy balance model [46]. Besides, our results highlight the nonlinear effect of precipitation decrease on glacier sensitivity to temperature, with a relatively larger impact associated with much drier conditions.

## 4 Climate and glaciation during MIS 3 interstadials and stadials

### 4.1 Climatic change

**Fig. 3:**
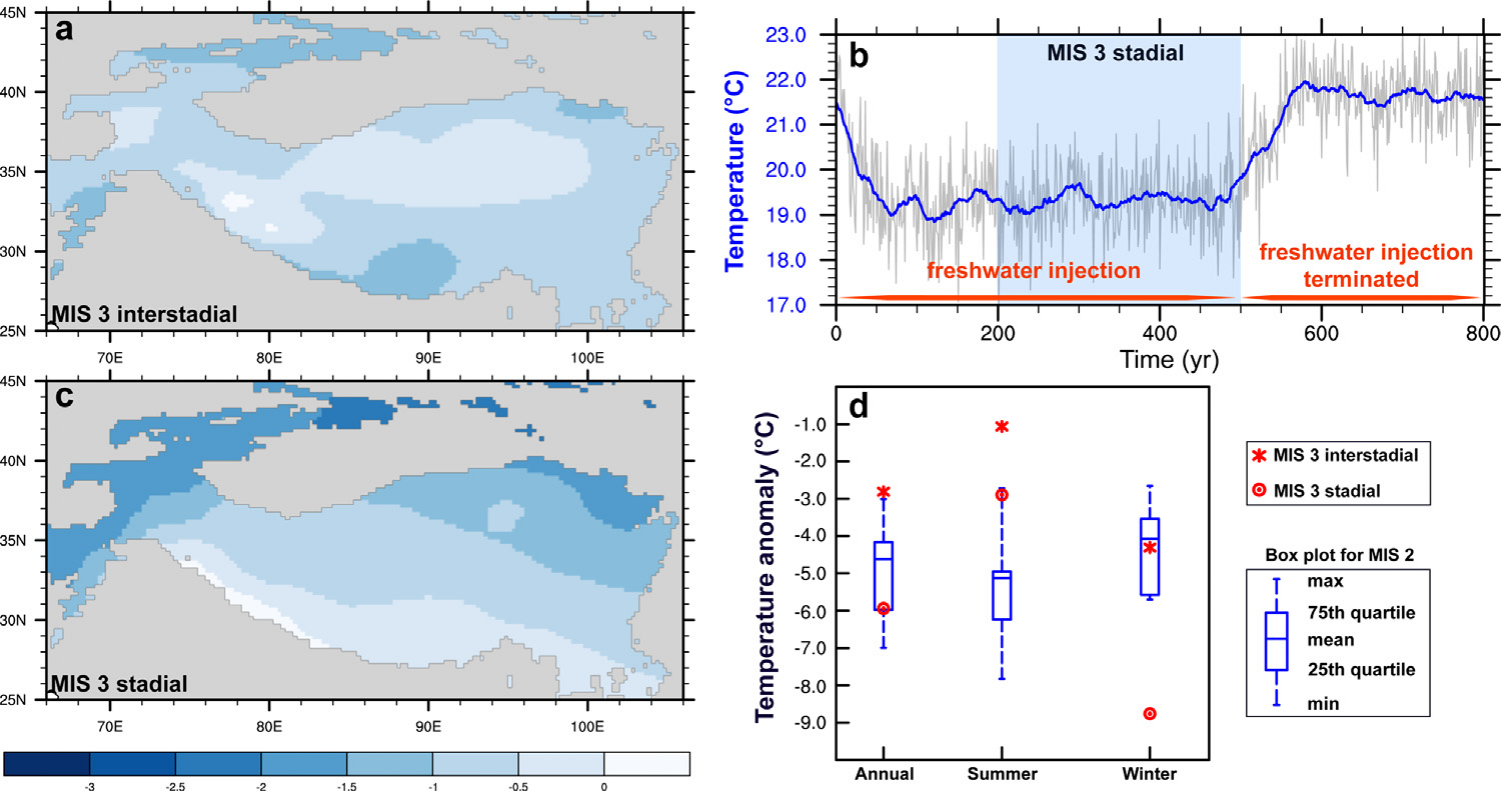
(a) Difference in summer temperature (°C) between the MIS 3 interstadial and pre-industrial simulations. (b) The evolution of summer temperature (°C) averaged over the Tian Shan in response to freshwater injection and the subsequent termination. The thick blue line shows the 31-yr running mean. (c) The difference in summer temperature (°C) between the MIS 3 stadial and pre-industrial simulations. (d) Boxplot for temperature anomaly (°C) averaged over the Tian Shan during MIS 2 relative to the pre-industrial based on PMIP models. The asterisk and circle represent the modeled temperature change during MIS 3 interstadial and stadial simulations, respectively.

Summer temperature decreases uniformly over the Tian Shan during the MIS 3 interstadial experiment relative to the preindustrial based on the NorESM1-F (Fig. 3a), with a decrease of regional mean temperature by ∼1.1 °C, larger than the temperature reduction (∼0.6 °C) averaged across the Tibetan Plateau. In response to freshwater injection and the associated weakened Atlantic Meridional Overturning Circulation (AMOC), there is a strong centennial-scale cooling leading to the formation of a stadial condition during MIS 3 (Fig. 3b and c), when the cooling magnitude in summer increases to ∼2.9 °C over the Tian Shan. Compared with the results from the PMIP models, it seems likely that summer temperature is higher during the MIS 3 interstadial than during MIS 2 (Fig. 3d), which is attributed to the relatively larger CO_2_ concentration and higher summer insolation during the MIS 3 interstadial at 38 ka. During the winter season with reduced insolation, the temperature averaged over the Tian Shan is reduced by ∼4.3 °C during the MIS 3 interstadial experiment than the preindustrial, close to the cooling magnitude (∼4.1 °C) during MIS 2, whereas it is ∼8.8 °C lower during the MIS 3 stadial experiment (Fig. 3d). In terms of annual mean temperature, the Tian Shan experiences a relatively warm climate during the MIS 3 interstadial experiment relative to MIS 2 (Fig. 3d) and a colder climate during the MIS3 stadial experiment (although the modeled temperature decrease is within the range of individual PMIP models).

**Fig. 4:**
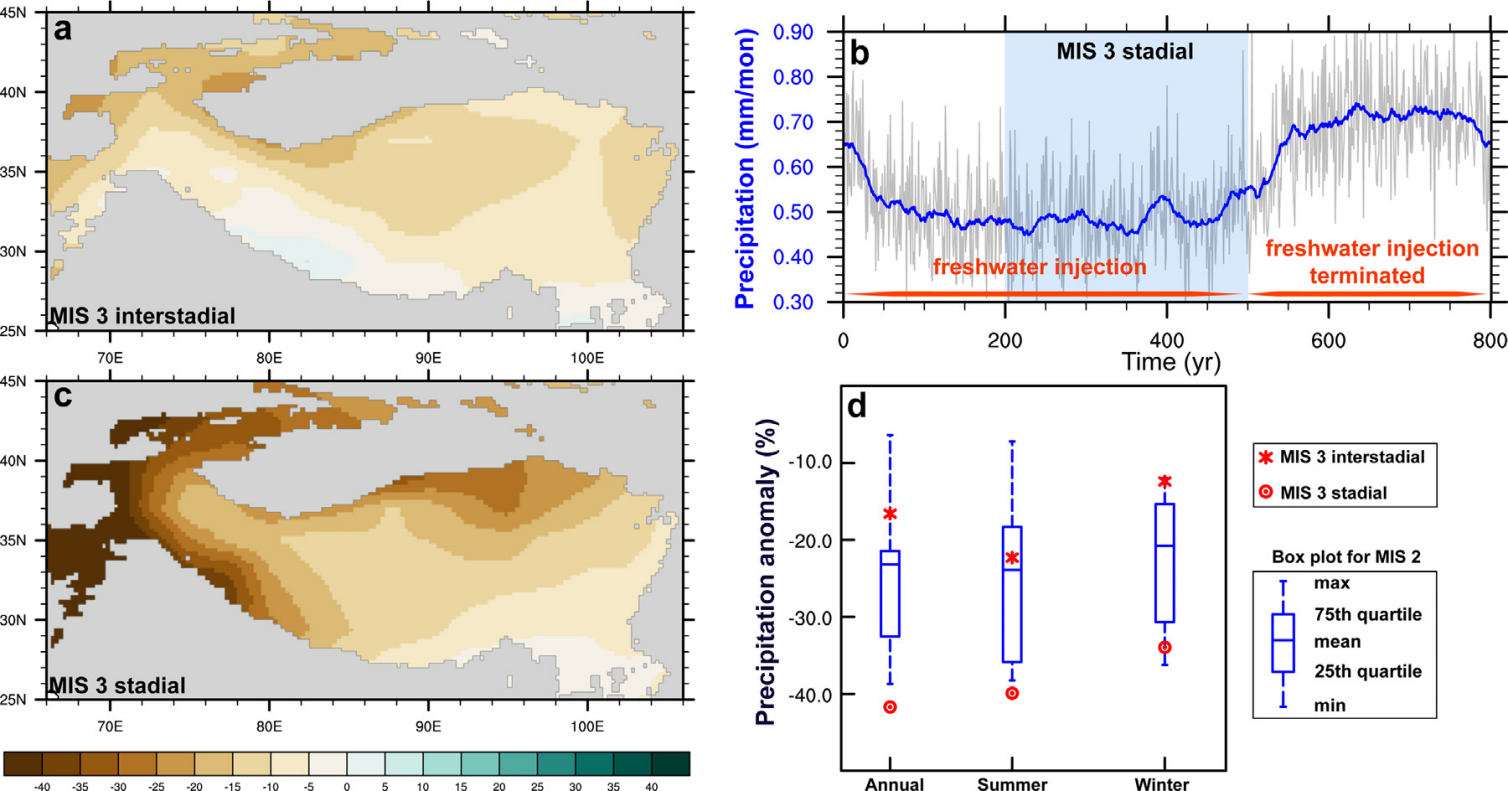
(a) Difference in annual mean precipitation (%) between the MIS 3 interstadial and pre-industrial simulations. (b) The evolution of annual mean precipitation (mm/month) averaged over the Tian Shan in response to freshwater injection and the subsequent termination. The thick blue line shows the 31-yr running mean. (c) The difference in annual mean precipitation (%) between the MIS 3 stadial and pre-industrial simulations. (d) Boxplot for precipitation anomaly (%) averaged over the Tian Shan during MIS 2 relative to the pre-industrial based on PMIP models. The asterisk and circle represent the modeled precipitation change during MIS 3 interstadial and stadial simulations, respectively.

The NorESM1-F predicts drier conditions over the Tian Shan and the Tibetan Plateau during the MIS 3 interstadial experiment relative to the preindustrial (Fig. 4a), with a decrease of annual mean precipitation by ∼17% and 9%, respectively. The reduced precipitation over the Tian Shan is linked with the weakened mid-latitudes westerlies during the MIS 3 interstadial experiment (Fig. S5) and is also observed in summer and winter seasons (Fig. 4d). During the MIS 3 stadial states, the Tian Shan becomes drier as the climate further cools, with a ∼42% decrease in annual mean precipitation (Fig. 4b and c). Compared with the multi-model ensemble mean of PMIP models, the climate tends to be relatively wetter over the Tian Shan during the MIS 3 interstadials than the MIS 2, whereas it is drier during the MIS 3 stadials than the MIS 2 in our simulations (Fig. 4d).

### 4.2 Glacier response

**Fig. 5:**
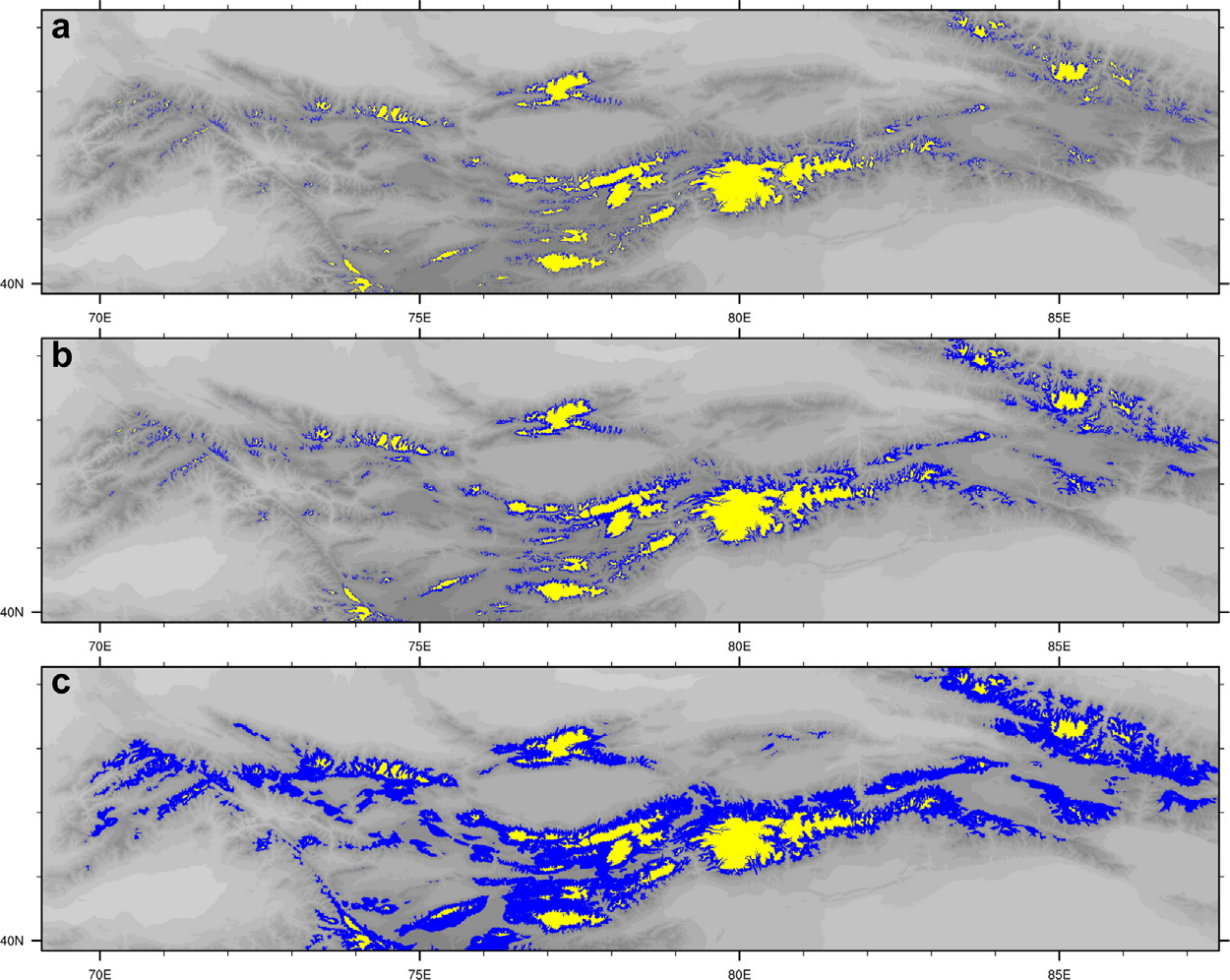
**Glacier distribution (blue shadings) over the Tian Shan during (a) MIS 3 interstadial, (b) MIS 3 stadial, and (c) MIS 2 simulations.** The yellow shadings show the modeled modern glacier distribution. The grey shadings indicate the topography.

Under the modeled colder and drier climate in the MIS 3 interstadial experiment, there are limited glacier expansions over the Tian Shan relative to the present day (Fig. 5a). The total glacier area increases by ∼36% to 24.8 × 10^3^ km^2^ during the MIS 3 interstadial experiment, with an ∆ELA of ∼75 m. The limited glacier advance results from the offset between the effect of temperature decrease (∼1.1 °C) and precipitation decrease (∼17%), consistent with the estimated glacier sensitivity under idealized scenarios (Fig. 2). During the MIS 3 stadial experiment, the enhanced cooling leads to additional glacier development (Fig. 5b), with a total glacier area of ∼36.8 × 10^3^ km^2^ (approximately twice as extensive as the present-day glacier cover) and an ELA of ∼3943 m (∆ELA = ∼162 m). However, the extent of glaciation is still restricted, largely owing to the much reduced precipitation during the MIS 3 stadial in our simulations. In contrast, significant glacier expansions are evident during MIS 2 in our simulations, with glaciation of high-altitude catchments and the growth of outlet glaciers into lower valleys (Fig. 5c). Glacier extent over the Tian Shan exhibits a five-fold increase during MIS 2 relative to the present, in tandem with an ∆ELA of ∼726 m.

**Fig. 6:**
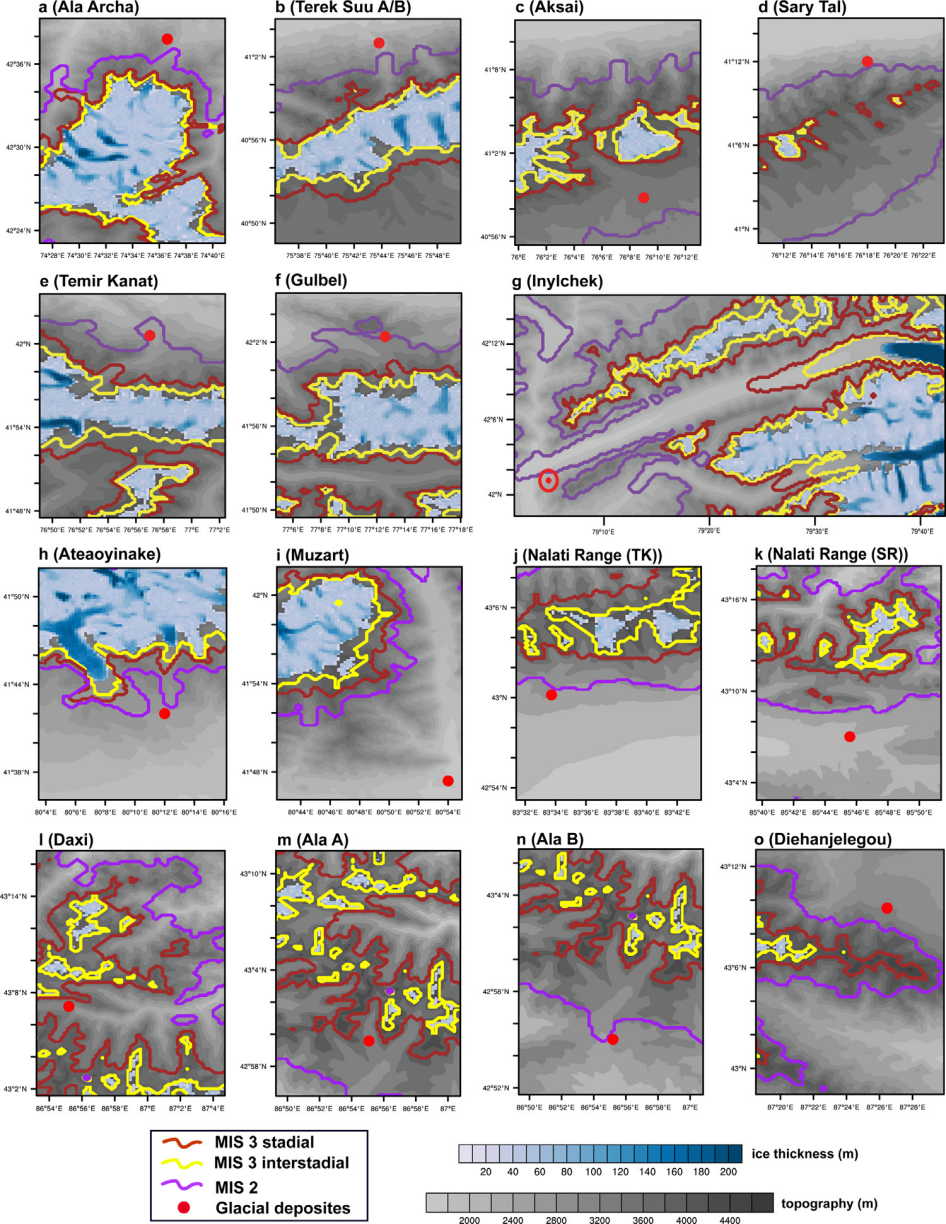
**Modeled glacier extent at the 15 sites (where a major MIS 3 glacier advance is proposed; Table S3) during the MIS 3 interstadial (yellow), MIS 3 stadial (brown), and MIS 2 (purple) simulations.** The grey label bar shows the topography (m) over the Tian Shan, and the blue label bar shows the modeled ice thickness in the present-day (m). See Fig. 1c for the location of each site.

**Fig. 7:**
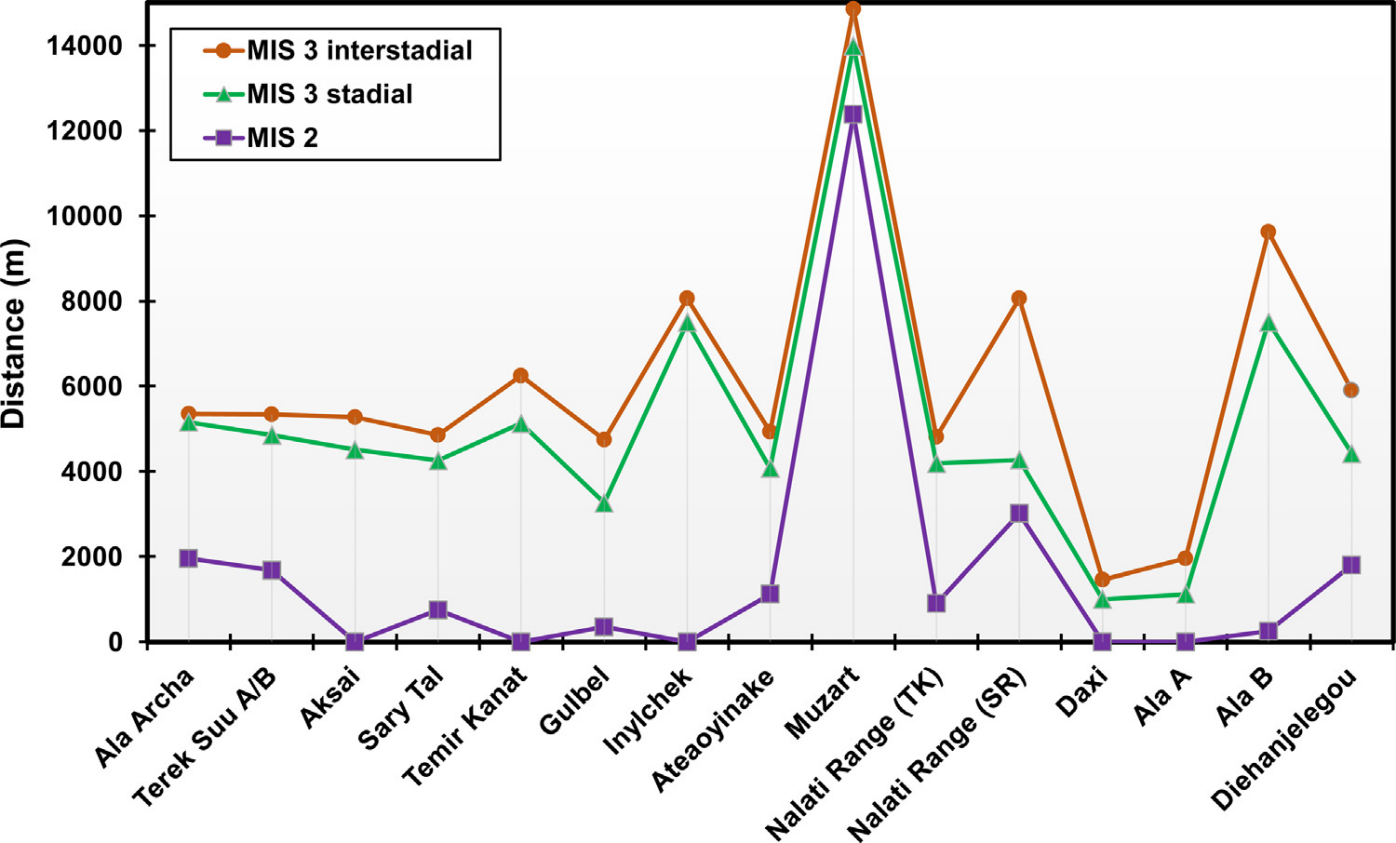
**The shortest distance (m) between the modeled glacier margin and the mapped “MIS 3” limits over the 15 sites (Table S3) during the MIS 3 interstadial (brown), MIS 3 stadial (green), and MIS 2 (purple) simulations.** See Fig. 1c for the location of each site.

Furthermore, the 250-m resolution of PISM allows us to perform a site-to-site comparison for glaciation over the Tian Shan. At the 15 sites, where glacial deposits were previously dated to MIS 3, our results indicate that the closest data-model match at all the sites is generally achieved under the MIS 2 climate conditions (Figs. 6 and 7). Specifically, at Aksai, Temir Kanat, Inylchek, Daxi, and Ala A, glacier expands and covers the locations where the “MIS 3” glacial deposits were found only during MIS 2 (Fig. 6), though the modeled glaciation is too extensive at Daxi and Ala A. In contrast, the model fails to capture the glacier advance at the Muzart in response to either MIS 2 or MIS 3 climate conditions (Fig. 6i), with the modeled ice margin being ∼12 (∼15) km away from the mapped moraines in the MIS 2 (3) experiment. For the remaining 9 sites, climate conditions during MIS 2 lead to the shortest distance between the modeled glacier margin and the mapped glacial deposits in our simulations, with a mean bias of ∼1300 m during MIS 2, ∼4700 m during the MIS 3 stadial, and ∼6000 m during the MIS 3 interstadial simulations (Fig. 7).

## 5 Conclusion and discussion

Given the ongoing debate on the widespread MIS 3 glaciation, from a perspective of climate-glaciation modeling, we assess the glacier sensitivity over the Tian Shan and examine the extent of glacier advances during MIS 3 and MIS 2. Our idealized results illustrate that the total glacier area shows an increasing rate of ∼18.7 × 10^3^ km^2^ for every 1 °C cooling, with an ∆ELA of ∼180 m/°C. For every 25% increase in precipitation, glacier extent increases by ∼6.1 × 10^3^ km^2^, and the ELA lowers by ∼76 m. However, a decrease in precipitation greatly reduces the sensitivity of glaciers to temperature decrease. During the MIS 3 interstadial, the model predicts a ∼1.1 °C summer cooling over the Tian Shan relative to pre-industrial, in tandem with reduced precipitation (∼17%). The climate gets much colder (–2.9 °C) and drier (–42%) during the MIS 3 stadial in response to the freshwater injection. Compared with MIS 2, summer temperature is higher during the MIS 3 interstadial and stadial simulations, while annual mean precipitation is relatively more (less) during the MIS 3 interstadial (stadial) simulations.

Under the colder and drier climate during the MIS 3 interstadial (stadial) simulations, the total glacier area over the Tian Shan increases by ∼36% (101%) relative to the present, with an ∆ELA of ∼75 m (162 m). However, the extent of MIS 3 glaciation is smaller than that during MIS 2, when there is a five-fold increase in glacier area and an ∆ELA of ∼726 m. Furthermore, through a site-to-site model-data comparison for glaciation, we demonstrate that climate conditions during MIS 2 lead to the shortest distance between the modeled glacier margin and the mapped glacial deposits previously considered to be MIS 3. These results suggest more extensive glacier advances over the Tian Shan during MIS 2 than MIS 3 on a regional scale, highlighting general synchronicity with the timing of maximum Northern Hemisphere ice sheets.

From a different perspective, our glaciation simulations provide important clues on the regional climate conditions that may yield a glacier extent close to the previously argued MIS 3 glacier margins. Based on our modeling results, a summer cooling of ∼5 °C with a ∼20% decrease in precipitation produces the shortest distance to the geologically reconstructed “MIS 3” limits. This is consistent with the results from the idealized sensitivity experiments, in which a temperature reduction of ∼5 °C with a 25% precipitation decrease gives rise to the closest match in many sites (Fig. S6). These results imply that putting aside the period we consider, a cooling of ∼5 °C with decreased precipitation is generally favorable for the expansion of glaciers to the mapped “MIS 3” glacial deposits across the Tian Shan. Although quantitative climatic reconstructions over the Tian Shan for MIS 2 and 3 are scarce, the inferred climatic perturbations are generally consistent with the geologic evidence across the Tibetan Plateau during MIS 2, suggesting a ∼5 °C cooling with ∼50% decrease in precipitation [47].

There are, however, limitations to be considered. The interstadial and stadial climates modeled here may represent two end members of the complex MIS 3 climate, characterized by multiple millennial-scale warm-to-cold transitions. As there is a lack of quantitative proxies for temperature and precipitation over the Tian Shan during MIS 3, the model results are hard to constrain directly by geologic evidence but are generally within the range of a few other MIS 3 simulations [48,49]. Nevertheless, we perform a set of idealized sensitivity experiments (Table S4) to explore the potential influences of uncertainties in climatic change and PISM parameters (Text S2). Our results demonstrate that although these uncertainties significantly impact the modeled extent of glaciations, glacier advances remain more extensive during MIS 2 than MIS 3 (Fig. S7). To further test our results and reduce the uncertainty in simulations, it is meaningful to organize and perform multimodel (including climate and glacier models) runs for MIS 3 in the future. In addition, our MIS 3 simulations are based on orbital parameters at 38 ka, and hence interstadial and stadial simulations with different orbital parameters across MIS 3 may affect the quantitative results reported here. However, using a low-resolution of the NorESM with varying orbital parameters (33, 37, 41, 48, and 56 ka) across MIS 3, the previous study confirms more extensive glaciation during MIS 2 than MIS 3 over the Tian Shan [50]. Our results highlight that a widespread regional glaciation over the Tian Shan may occur during MIS 2 rather than MIS 3. Still, our modeling does not exclude the possibility of individual glacier advances during MIS 3. This is because (i) the NorESM1-F model used here is insufficient to depict the microclimatic variations within individual mountain ranges and (ii) the physical properties of individual glaciers may vary across the Tian Shan. Besides, a near-synchronous glaciation over the Tian Shan with the Northern Hemisphere ice sheets might not be widely applicable to cold, sub-freezing arid regions in Central Asia (i.e., sublimation-dominated glaciers; [22]), as the climate sensitivity of glaciers differs substantially [29].

Nevertheless, for the first time, we present a quantitative reconstruction of glacier extent over the Tian Shan during MIS 3 by combining a 250-m ice sheet model and a global climate model. Our results highlight more extensive glaciation during MIS 2 relative to MIS 3 over the Tian Shan on a regional scale. These results aid in resolving the ongoing debate on the widespread “MIS 3” glaciation (together with additional field studies across the Tian Shan using multiple dating techniques) and in advancing our understanding of the timing and style of Quaternary glaciation across Central Asia. Furthermore, our study provides a framework to resolve potential data-data controversy on the timing of glaciation in other periods and other areas of the world.

## Declaration of competing interest

The authors declare that they have no conflicts of interest in this work.

## Appendix Supplementary materials

Image, application 1
