# Supplementary material for: Widespread glacier advances across the Tian Shan during Marine Isotope Stage 3 not supported by climate-glaciation simulations
Source: Fundam Res. 2022 Mar 2;3(1):102–10. doi: 10.1016/j.fmre.2022.01.033 (PMC11197723; doi:10.1016/j.fmre.2022.01.033)
Supplement: Supplementary file 1 [file mmc1.pdf]

Supplementary Information for

Widespread glacier advances across the Tian Shan during Marine Isotope Stage 3 not supported by climate-glaciation simulations

Qing Yan*, Lewis A. Owen, Chuncheng Guo, Zhongshi Zhang, Jinzhe Zhang, Huijun Wang

Text S1. The Parallel Ice Sheet Model

In this study, we utilize the Parallel Ice Sheet Model (PISM; version 1.2.1) to investigate the response of glaciers over the Tian Shan to past climatic change. The PISM is a three dimensional, thermodynamically coupled, hybrid ice sheet model that has been widely used in modeling ice evolution at various scales during the past, present, and future climates. In PISM, surface mass balance is defined as the difference between accumulation and ablation. Accumulation depends on the monthly precipitation and temperature, which is identical to precipitation when air temperature is below 0 °C and is linearly reduced to zero when air temperature increases above 2 °C. Surface ablation is estimated using the positive degree-day scheme. The melt factor for snow (ice) is set to 4 (12) mm water equivalent d^{-1} $°C^{-1}$, which is uniformly applied over the entire model domain and does not vary with temperature. The standard deviation of surface air temperature is spatially varied across Tian Shan and derived from the High Asia Refined analysis version 2. Meantime, melted snow and ice are allowed to become superimposed ice by a constant refreeze fraction (30%).

For ice thermodynamics, a "hybrid" approximation of the Stokes model (i.e., SIA+SSA) is adopted in PISM so that the roles of vertical deformation and longitudinal stretching in the deformation of ice are taken into account. The hybrid scheme provides a consistent treatment for different flow regimes and leads to better descriptions of ice streams and fast-flowing outlet glaciers. The enhancement factor for SIA and SSA is set to 6 and 0.9, respectively. An enthalpy-based scheme is employed in PISM, which guarantees energy conservation even when the temperature is at the pressure-melting point. Moreover, this scheme accounts for melting and refreezing processes in temperate ice, allowing polythermal and fully-temperate glaciers to be modeled. Based on the hypothesis that ice is underlain by a layer of till, the PISM adopts a "pseudo-plastic" law that relates ice base velocity to the basal shear stress to estimate basal sliding:

$$\tau_b = -\tau_c \frac{u_b}{u_0^q \left|u_b\right|^{1-q}}$$

where q is the sliding exponent and is set to 0.9, τ_b is the basal shear stress (tangential sliding), u_b is the sliding velocity, u_0 is the velocity threshold (100 m/year), and τ_c is the yield stress that is defined as:

$$\tau_c = \tan(\phi) N_{\text{till}}$$

where ϕ is the till friction angle that is a function of bed elevation and N_{till} is the effective pressure that is determined by the overburden pressure and the fraction of effective water thickness in the till.

Additionally, the PISM uses the modified flat earth elastic lithosphere relaxing asthenosphere model to incorporate the effect of ice load on vertical earth deformation. Additional information regarding the PISM can be found in the PISM online manual (https://www.pism.io/docs/).

Text S2. Sensitivity experiments for MIS 3 glaciations

The uncertainties in the climate model and ice sheet model could introduce biases in the modeled extent of glaciations over the Tian Shan. However, owing to the scarce of quantitative climate proxies and the reconstructions regarding glacier physical properties, it remains difficult to constrain the modeled climatic change and PISM parameters during MIS 3 over the Tian Shan. Here, we perform the following sensitivity experiments (Table S4) to examine the uncertainty of the modeled ice extent induced by temperature, precipitation, and PISM parameters.

(i) Influence of ice sheet model parameters

As there is little data to constrain ice sheet model parameters during MIS 3 and 2, we use the same model parameters as the control run in the experiments regarding glacier behavior during MIS 3 and 2. To test the influence of ice sheet model parameters, we rerun the MIS 3 interstadial and stadial experiments, but with the melt factor for ice and snow (a key factor for surface mass balance) reduced by 50%. This indicates an idealized condition favorable for glaciations during MIS 3, though not supported by geological evidence.

(ii) Influence of precipitation

The inferred “MIS 3” glacier expansions in the Tian Shan have been attributed to significantly wetter conditions at that time, at least relative to MIS 2. However, geologic evidence directly from the Tian Shan indicates extremely drier conditions during MIS 3 and 2 than the present, which is also seen in our simulations. To test the influence of precipitation, we rerun the MIS 3 interstadial and stadial experiments but using modern precipitation at this time. This indicates a higher precipitation scenario and hence favorable condition for glaciations during MIS 3, though opposite to multi-proxies over the Tian Shan.

(iii) Influence of temperature

Given the scarce of quantitative temperature reconstructions for MIS 3 over the Tian Shan, the amplitude of cooling in the simulations remains hard to constrain directly by geological evidence. To test the influence of temperature, we rerun the MIS 3 interstadial and stadial experiments but with an additional 2°C cooling. In these sensitivity experiments, the annual cooling over the Tian Shan reaches ~5°C and 8°C during the MIS 3 interstadial and stadial, respectively, generally covering the cooling amplitude seen in the Guliya ice core during MIS 3 (despite chronology uncertainty). This indicates a colder scenario and hence favorable condition for glaciations during MIS 3.

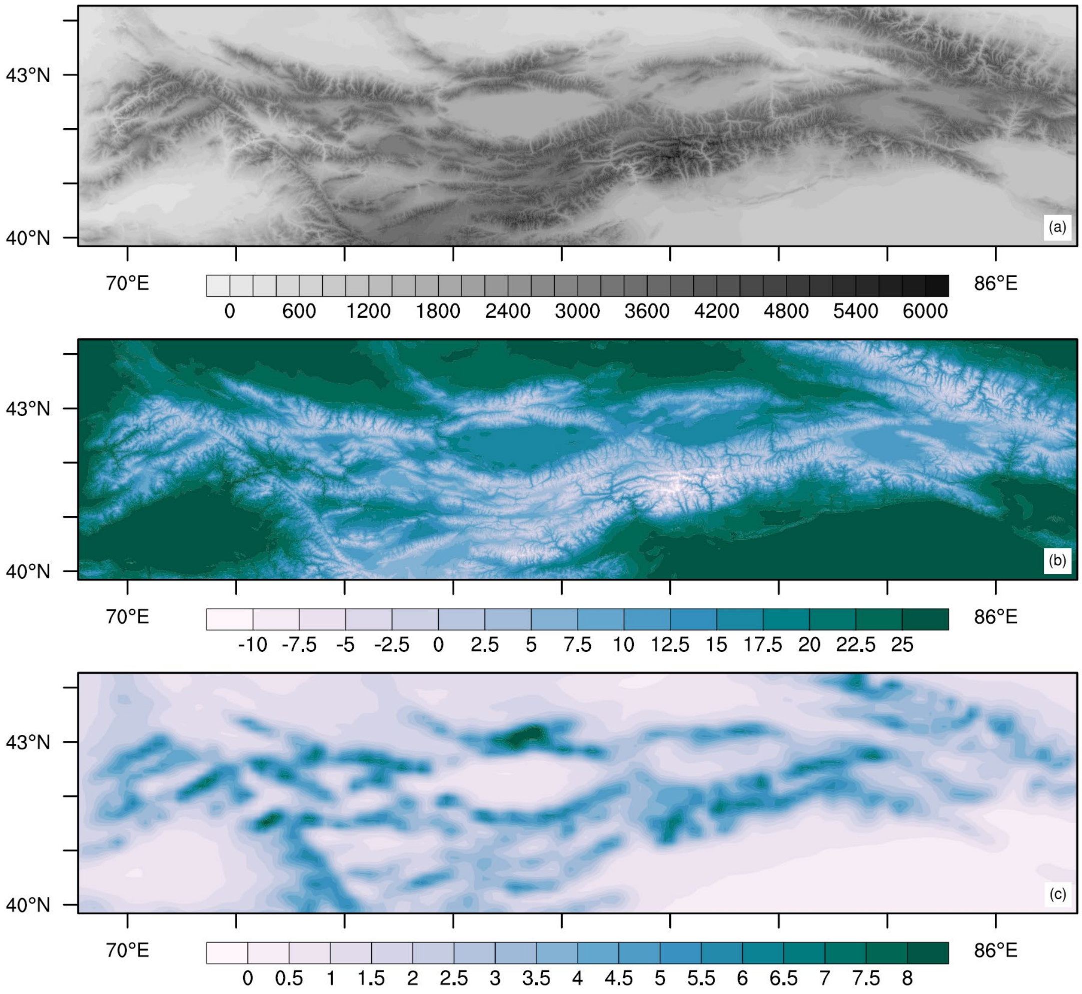

Figure S1. Boundary conditions in the present day used in the PISM. (a) Topography (m), (b) summer temperature (°C), and (c) annual mean precipitation (mm/day).

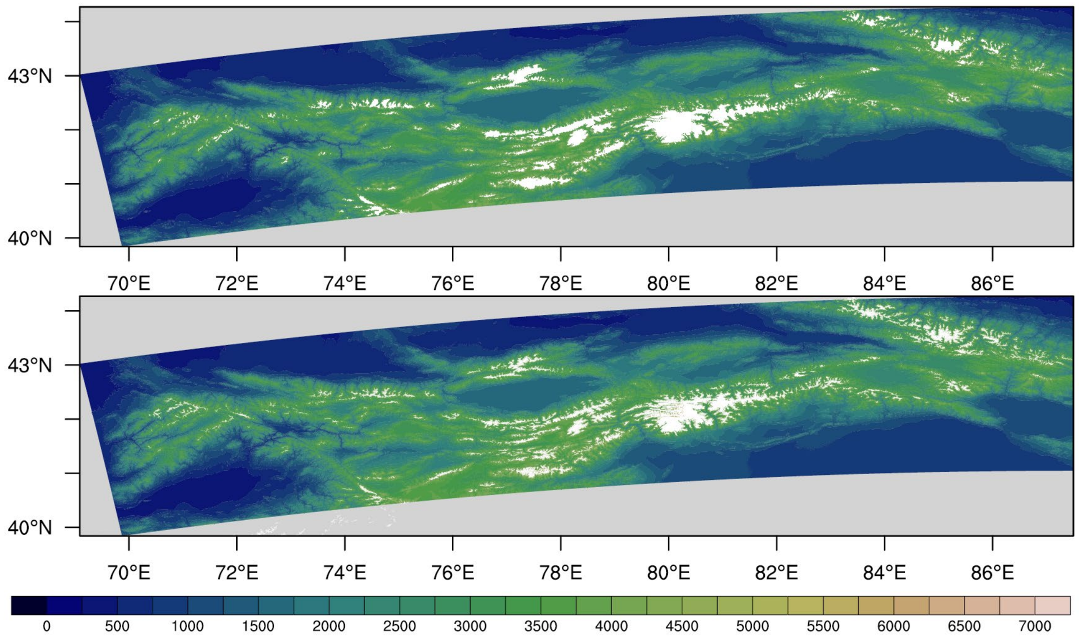

Figure S2. Modern glacier distribution (white shadings) in the (top) PISM simulation and (bottom) observations.

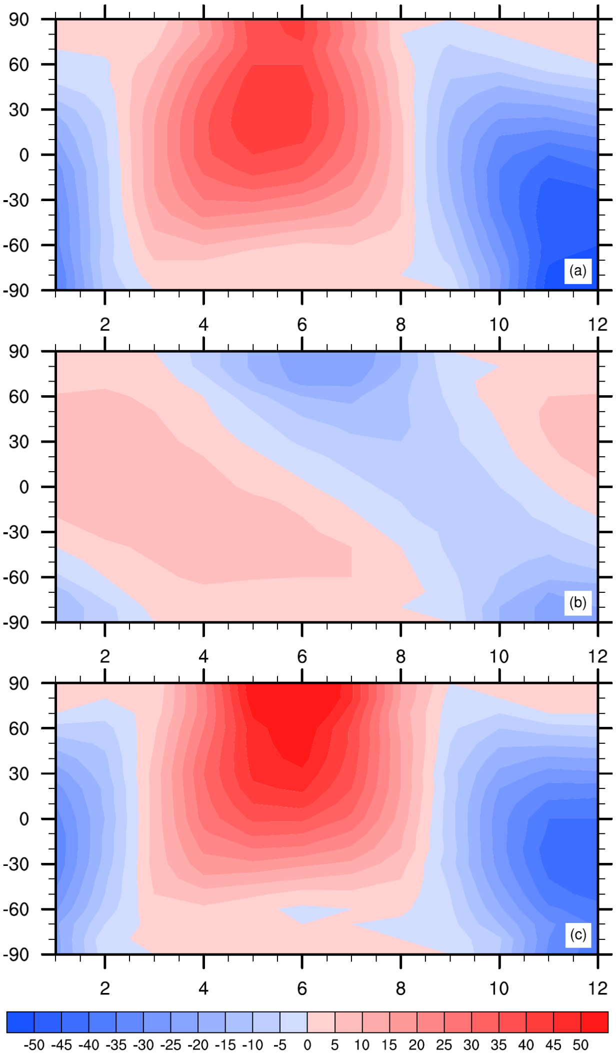

Figure S3. Insolation anomalies (W m^{-2}) at (b) 38 ka and (b) 21 ka relative to present based on Berger and Loutre (1991). (c) The difference (W m^{-2}) between 38 and 21 ka.

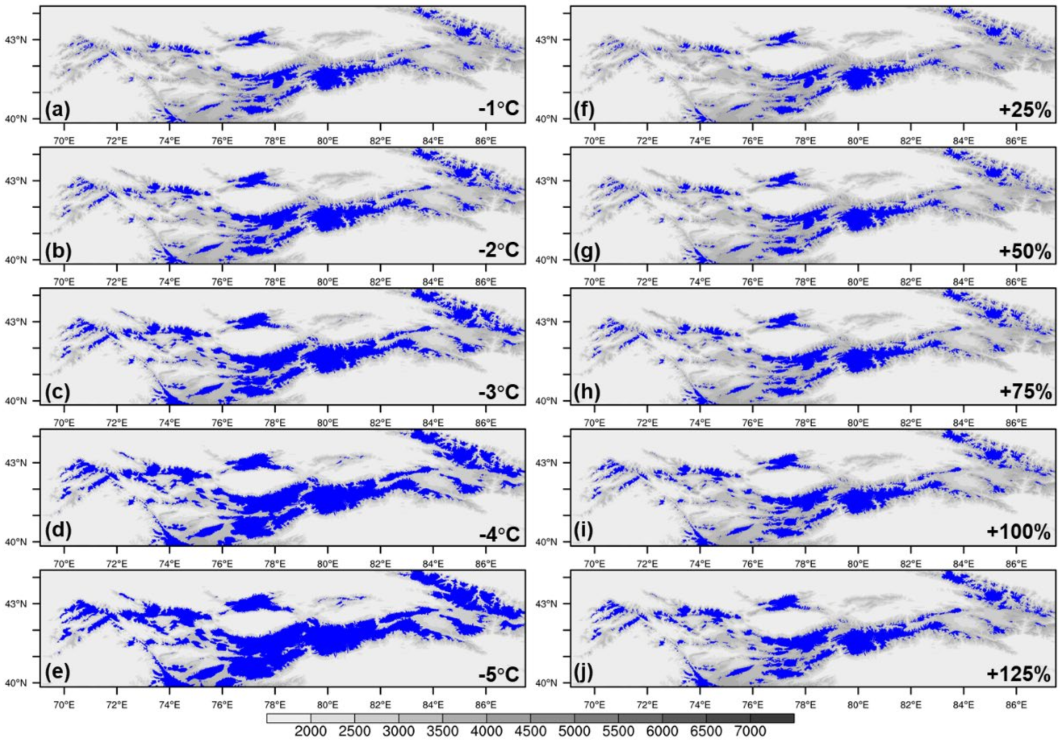

Figure S4. Glacier distribution (blue shadings) over the Tian Shan in response to (a−e) the imposed step cooling of −1 to −5.0°C under a modern precipitation regime and (f−j) the imposed step precipitation increases of +25% to +125% under a modern temperature regime.

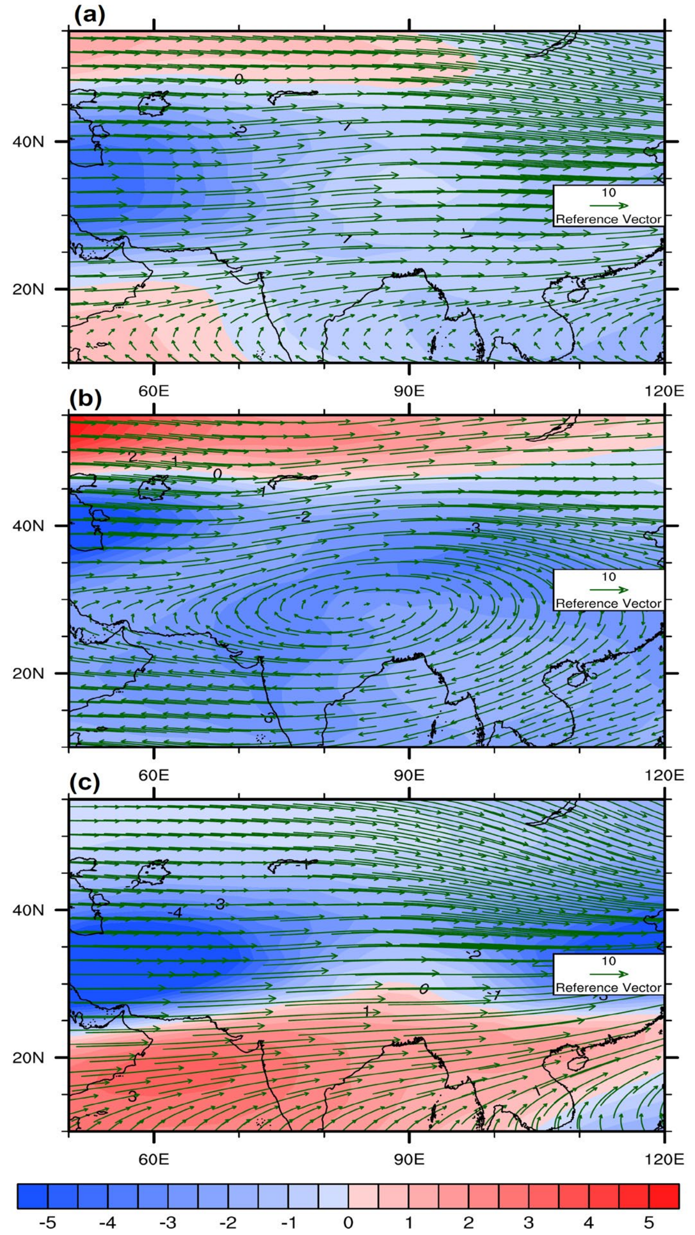

Figure S5. Wind fields (m/s; vectors) at 200 hPa at the preindustrial and the zonal wind anomaly (m/s; shadings) during MIS 3 interstadial simulation relative to the preindustrial for (a) annual mean, (b) summer season, and (c) winter season.

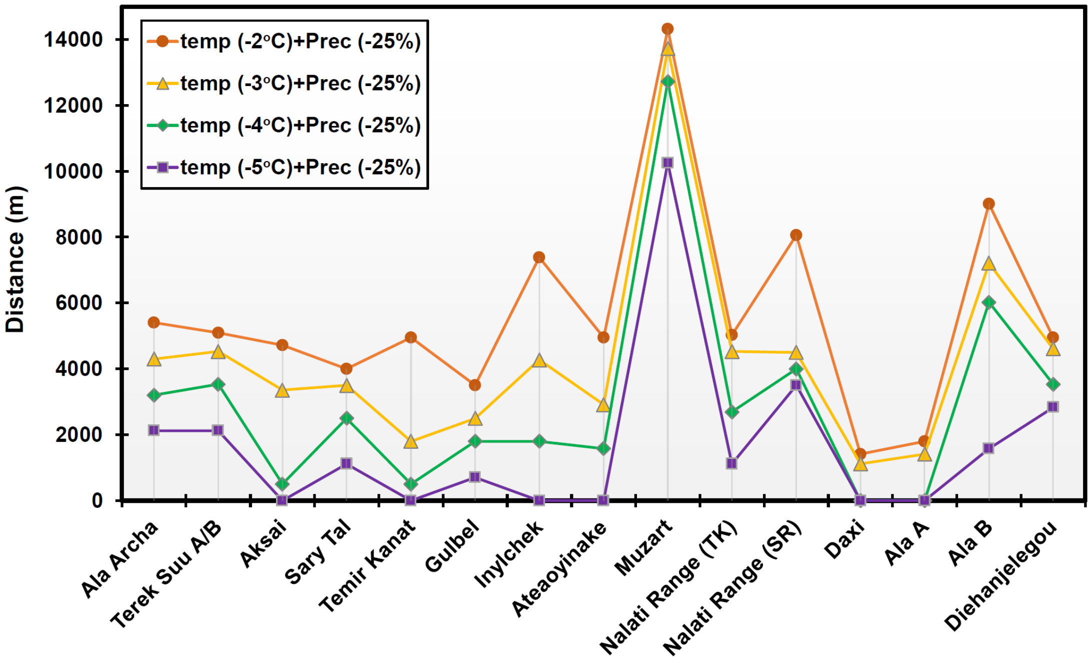

Figure S6. The shortest distance (m) between the modeled glacier margin and the mapped "MIS 3" limits over the 15 sites (Table S3) in the idealized experiments with a step cooling of −1 to −5.0°C under a –25% precipitation regime.

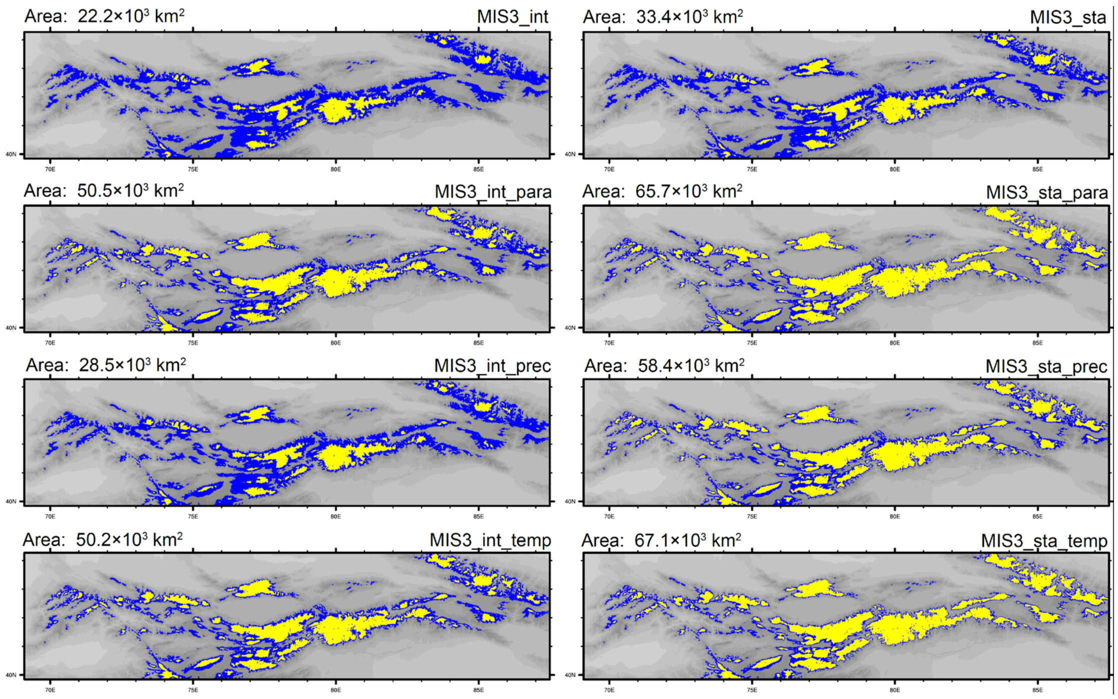

Figure S7. The modeled glacier extent in the MIS 3 sensitivity experiments (yellow shadings; Table S4) and during MIS 2 (blue shadings; 82.7×10^3 km^2) at 1-km resolution. The total glacier area in the MIS 3 sensitivity experiments is shown in the upper-left corner.

Table S1. Boundary conditions for the MIS 3 interstadial and pre-industrial experiments

	**MIS 3 interstadial**	**Pre-industrial**
**Eccentricity**	0.013676	0.016708
**Obliquity**	23.268	23.441
**Perihelion – 180°**	205.94	102.72
**CO_2 (ppm)**	215	285
**CH_4 (ppb)**	550	792
**N_2O (ppb)**	260	276
**CFC (ppt)**	0	12.5
**Solar constant (Wm^{-2})**	1360.9	1360.9
**Ice sheets**	Data-constrained at 38 ka	Modern
**Vegetation**	Modern	Modern

Table S2. Basic information on the PMIP models used in this study. More details can be found in https://pmip4.lsce.ipsl.fr/, https://pmip3.lsce.ipsl.fr/, and https://pmip2.lsce.ipsl.fr/

Model ID		Model type	Atmosphere resolution
Eight PMIP2 models			
1	CCSM3.0*	AOGCM	~2.8º × 2.8º
2	CNEM-CM3.3	AOGCM	~2.8º × 2.8º
3	ECBILTCLIO*	AOGCM	~5.6º × 5.6º
4	FGOALS-1.0g	AOGCM	~2.8º × 3–6º
5	HadCM3M2*	AOGCM	~3.75º × 2.5º
6	HadCM3M2-gvm*	AOVGCM	~3.75º × 2.5º
7	IPSL-CM4-V1-MR*	AOGCM	~3.75º × 2.5º
8	MIROC3.2	AOGCM	~2.8º × 2.8º
Nine PMIP3 models			
1	CCSM4*	AOGCM	~1.25º × 0.9º
2	CNEM-CM5	AOGCM	~1.4º × 1.4º
3	COSMOS-ASO	AOVGCM	~3.75º × 3.7º
4	FGOALS-g2	AOVGCM	~2.8º × 3–6º
5	GISS-E2-R*	AOGCM	2.5º × 2.0º
6	IPSL-CM5A-LR	AOVGCM	~3.75º × 1.9º
7	MIROC-ESM*	AOVGCM	~2.8º × 2.8º
8	MPI-ESM-P	AOGCM	~1.875º × 1.9º
9	MRI-CGCM3*	AOGCM	~1.175º × 1.1º
Four PMIP4 models			
1	AWI-ESM-1-1-LR	AOGCM	~1.875º × 1.875º
2	INM-CM4-8*	AOGCM	~1.5º × 2.0º
3	MIROC-ES2L	AOGCM	~2.8º × 2.8º
4	MPI-ESM1-2-LR	AOGCM	~1.875º × 1.875º

*These models can reproduce a colder and direr condition during MIS 2 relative to present over the Tian Shan.

Table S3. Brief information on the glacial deposits based on which a major MIS 3 glacier advance is proposed. CNE: cosmogenic nuclide exposure; OSL: optically stimulated luminescence; ESR: electron spin resonance. The majority of the chronological dataset is based on the recalculated ages from Gribenski et al. (2018).

ID	Sites	Moraine	Lat (°N)	Lon (°E)	Ages (ka)	Dating Methods	References
1	Ala Archa	MIII moraine	42.63	74.61	47.6±3.1	CNE	Koppes et al. (2008)
2	Terek Suu A/B	M2/MII/MIII moraine	41.05	75.73	31.6±1.9	CNE	Koppes et al. (2008)
					49.7±2.9	OSL	Narama et al. (2009)
					33.4±2.7	CNE	Koppes et al. (2008)
3	Aksai	MIIIb moraine	40.98	76.15	33.9±1.9	CNE	Koppes et al. (2008)
4	Sary Tal	MII moraine	~41.2	~76.3	31.5±2.7	OSL	Narama et al. (2009)
					24.3±1.9	OSL	
5	Temir Kanat	MI moraine	~42.01	~76.95	56.3±5.8	OSL	Narama et al. (2009)
					71.3±5.6	OSL	
6	Diehanjelegou	XCS101-106 moraine	~43.159	~87.440	38.6±3.6	CNE	Batbaatar et al. (2021)
					21.4±2	CNE	
					55.7±5.2	CNE	
					30.0±3.0	CNE	
					42.0±4.2	CNE	
					23.8±2.2	CNE	

7	Inylchek	Terminal moraine	42.019	79.079	39.1±2.6	CNE	Lifton et al. (2014)
8	Ateaoyinake	3rd moraine set	~41.69	~80.21	40.9±4	ESR	Zhao et al. (2009)
					46.2±4.2	ESR	
					51±4.8	ESR	
					54±5.2	ESR	
9	Muzart	5th set of Pochengzi moraine	~41.79	~80.91	39.5±4	ESR	Zhao et al. (2010)
					40.4±4	ESR	
10	Nalati Range	Takelete TK4	~42.99	~83.6	70.2±4	CNE	Zhang et al. (2016)
					33.1±2.1	CNE	
					50.1±3.1	CNE	
					63.5±3.8	CNE	
					39.6±2.6	CNE	
					51.6±3.0	CNE	
					55.5±3.4	CNE	
					37.5±2.2	CNE	
11	Nalati Range	Sairenwuxunsala SR4	43.1169	85.7596	29.5±1.7	CNE	Zhang et al. (2016)
					19.8±1.2	CNE	

					13.2±0.9	CNE	
					19.1±1.1	CNE	
12	Daxi	Shangwangfeng till	~43.12	~86.92	35.0±3.5	ESR	Zhao et al. (2006)
					27.6	ESR	Yi et al. (2001)
					37.4	ESR	Yi et al. (2001)
13	Ala A	M3 moraine	~42.99	~86.92	21.2±1.3	CNE	Li et al. (2014)
					23.7±1.4	CNE	
					25.7±1.5	CNE	
					24.6±1.4	CNE	
					37.9±2.1	CNE	
					29.6±1.7	CNE	
					30.3±1.9	CNE	
14	Ala B	M4 moraine	~42.92	~86.92	48.3±2.8	CNE	Li et al. (2014)
					41.7±2.5	CNE	
					44.0±2.5	CNE	
					36.7±2.1	CNE	
					44.8±2.7	CNE	

					76.9±4.3	CNE	
					34.3±2.7	CNE	
15	Gulbel	UNIT II moraine	~42.04	~77.21	40.5±3.1	CNE	Koppes et al. (2008)
					32.4±2.4	CNE	
					30.0±2.3	CNE	
					41.9±3.3	CNE	
					98.1±7.9	CNE	

Table S4. Sensitivity experiments for MIS 3 glaciations.

Experiments	Temperature	Precipitation	Positive-degree day factors	Notes
MIS3_int	MIS3 interstadial	MIS3 interstadial	as control run	/
MIS3_sta	MIS3 stadial	MIS3 stadial	as control run	/
MIS3_int_para	MIS3 interstadial	MIS3 interstadial	reduced by 50%	favorable for glaciation
MIS3_sta_para	MIS3 stadial	MIS3 stadial	reduced by 50%	favorable for glaciation
MIS3_int_prec	MIS3 interstadial	Modern	as control run	wetter scenario
MIS3_sta_prec	MIS3 stadial	Modern	as control run	wetter scenario
MIS3_int_temp	MIS3 interstadial + 2°C cooling	MIS3 interstadial	as control run	colder scenario
MIS3_sta_temp	MIS3 stadial+ 2°C cooling	MIS3 stadial	as control run	colder scenario
